# β-Catenin is required for the differentiation of iNKT2 and iNKT17 cells that augment IL-25-dependent lung inflammation

**DOI:** 10.1186/s12865-015-0121-0

**Published:** 2015-10-19

**Authors:** Rosa Berga-Bolaños, Archna Sharma, Farrah C. Steinke, Kalyani Pyaram, Yeung-Hyen Kim, Dil A. Sultana, Jessie X. Fang, Cheong-Hee Chang, Hai-Hui Xue, Nicola M. Heller, Jyoti Misra Sen

**Affiliations:** Immune Cells and Inflammation Section, National Institute on Aging, National Institutes of Health, Baltimore, MD 21224 USA; Department of Microbiology, Interdisciplinary Immunology Graduate Program, University of Iowa, Iowa City, IA 52242 USA; Department of Microbiology and Immunology, The University of Michigan Medical School, Ann Arbor, MI 48109 USA; Department of Anesthesiology and Critical Care Medicine, The Johns Hopkins University School of Medicine, Baltimore, MD 21287 USA; Department of Medicine, The Johns Hopkins University School of Medicine, Baltimore, MD 21287 USA; National Institute on Aging, NIH, Baltimore, MD 21224 USA; Present addresses: Center for Translational Research, The Feinstein Institute for Medical Research, 350 Community Dr., Manhasset, NY 11030 USA; Present addresses: Center for Immunology and Microbial Disease, Albany Medical College, Albany, NY 12208 USA

**Keywords:** Beta-catenin, iNKT cells, iNKT2, iNKT17, IL-17RB

## Abstract

**Background:**

Invariant Natural Killer T (iNKT) cells have been implicated in lung inflammation in humans and also shown to be a key cell type in inducing allergic lung inflammation in mouse models. iNKT cells differentiate and acquire functional characteristics during development in the thymus. However, the correlation between development of iNKT cells in the thymus and role in lung inflammation remains unknown. In addition, transcriptional control of differentiation of iNKT cells into iNKT cell effector subsets in the thymus during development is also unclear. In this report we show that β-catenin dependent mechanisms direct differentiation of iNKT2 and iNKT17 subsets but not iNKT1 cells.

**Methods:**

To study the role for β-catenin in lung inflammation we utilize mice with conditional deletion and enforced expression of β-catenin in a well-established mouse model for IL-25-dependen lung inflammation.

**Results:**

Specifically, we demonstrate that conditional deletion of β-catenin permitted development of mature iNKT1 cells while impeding maturation of iNKT2 and 17 cells. A role for β-catenin expression in promoting iNKT2 and iNKT17 subsets was confirmed when we noted that enforced transgenic expression of β-catenin in iNKT cell precursors enhanced the frequency and number of iNKT2 and iNKT17 cells at the cost of iNKT1 cells. This effect of expression of β-catenin in iNKT cell precursors was cell autonomous. Furthermore, iNKT2 cells acquired greater capability to produce type-2 cytokines when β-catenin expression was enhanced.

**Discussion:**

This report shows that β-catenin deficiency resulted in a profound decrease in iNKT2 and iNKT17 subsets of iNKT cells whereas iNKT1 cells developed normally. By contrast, enforced expression of β-catenin promoted the development of iNKT2 and iNKT17 cells. It was important to note that the majority of iNKT cells in the thymus of C57BL/6 mice were iNKT1 cells and enforced expression of β-catenin altered the pattern to iNKT2 and iNKT17 cells suggesting that β-catenin may be a major factor in the distinct pathways that critically direct differentiation of iNKT effector subsets.

**Conclusions:**

Thus, we demonstrate that β-catenin expression in iNKT cell precursors promotes differentiation toward iNKT2 and iNKT17 effector subsets and supports enhanced capacity to produce type 2 and 17 cytokines which in turn augment lung inflammation in mice.

## Background

Invariant Natural killer T (iNKT) cells participate in both innate and adaptive immune functions [1–4]. iNKT cells develop from CD4^+^ CD8^+^ double positive (DP) thymocytes that express an invariant T-cell receptor (TCR). In mice TCRα chain Vα14-Jα18 pairs with Vβ8, Vβ7 or Vβ2 to generate a TCR repertoire that recognizes glycolipid antigens presented by the MHC class I-like molecule CD1d [5–7]. Recognition of lipid antigens presented by CD1d expressed on DP thymocytes leads to commitment to the iNKT lineage. Immature iNKT0 cells then receive developmental cues that control differentiation into type1 iNKT1, type2 iNKT2 and type17 iNKT17 cells that produce cytokines that are similar to those produced by mature effector CD4 T cells after encounter with antigen [8–10]. However, in contrast to effector CD4 T cells, iNKT cells gain the ability to express cytokines during development and respond rapidly when stimulated [1, 11]. Whereas the specific transcription factors that regulate cytokine production by each subset of iNKT cells are well defined, the transcriptional programs that control iNKT cell generation and differentiation into effector cells remain to be delineated.

The connection between iNKT cells and the development of asthma or allergic lung inflammation in mice was revealed and non-adaptive immune cellular candidates were sought to understand how non-allergic environmental triggers could lead to asthma. Typical allergen sensitization and challenge models could not explain why air pollution, viruses and obesity enhanced the disease. These studies uncovered roles for iNKT and innate lymphoid cell populations in the lung in modulating allergic lung inflammation. Since then, numerous human studies have attempted to correlate frequency, function and location of iNKT cells to asthma severity. Akbari et al. showed that lung CD4+ iNKT cells from asthmatics secreted both IL-4 and IL-13 but little IFN-γ on αGalCer stimulation [12]. However, conclusions about the contribution of peripheral blood iNKT cells from asthmatics to the severity of asthma have been equivocal [13–16]. Nonetheless, iNKT cells play an essential role in viral and pediatric exacerbations of asthma and in severe asthma [17]. Defining their contribution to allergic lung inflammation is of critical clinical importance to finding novel approaches to regulate the number and function of iNKT cells. Furthermore, understanding the molecular mechanisms that regulate how iNKT cells develop and differentiate is key to controlling the number and frequency of these cells.

β-Catenin is ubiquitously expressed and in T cells is augmented in response to TCR signals and regulates T cell development in the thymus [18–23]. In addition to conventional T cells, the thymus is also the site for innate immune cell development [24, 25]. We have demonstrated that β-catenin expression regulates the generation of innate-like CD8 (iCD8) thymocytes [26]. However, a role for β-catenin in the generation and differentiation of iNKT cells in the thymus remains to be defined.

In this study we report that mice with enforced expression of β-catenin in iNKT cell precursors (β-CAT-Tg) experience augmented IL-25-dependent lung inflammation. iNKT cells in the lung produced increased amounts of IL-13 and IL-17 and presented intensified inflammation at alveolar sites and surrounding blood vessels. We demonstrate that augmented lung inflammation correlated with enhanced generation of iNKT2 and iNKT17 cells in β-CAT-Tg thymus at the expense of iNKT1 cells. Using mice in which β-catenin was deleted in iNKT cell precursors (β-CAT-KO) we show that expression of β-catenin is selectively required for differentiation of iNKT2 and iNKT17 cells as iNKT1 cells developed normally. In conclusion, this study demonstrates that β-catenin expression was required to promote differentiation and functional maturation of iNKT2 and iNKT17 cells that contributed to augmented IL-25-dependent lung inflammation.

## Methods

### Mice

β-CAT-Tg mice, expressing an activated form of β-catenin in thymocytes and T cells from the proximal *Lck* promoter, have been previously described [27]. β-CAT-cKO mice were generated by breeding mice bearing a LoxP-flanked gene encoding β-catenin (β-CAT^flox/flox^) [28] with mice expressing the Cre recombinase under the control of the *Cd4* promoter (CD4-Cre mice). All the mice used are on a C57BL/6 genetic background. CD45.1+ C57BL/6.SJL mice were purchased from Taconic. CD45.1 + 2+ mice were generated by breeding C57BL/6.SJL mice with C57BL/6 mice. Age-matched (7–10 weeks old) littermate controls or C57BL/6 mice were used in all experiments. All mice were bred and maintained in animal facility at the National Institute on Aging (NIA). The studies were carried out in accordance with the recommendations in the Guide for the Care and Use of Laboratory Animals (NRC 2010). The protocol was approved by the Animal Care and Use Committee of the NIA Intramural Research Program, NIH. This program is fully accredited by the Association for Assessment and Accreditation of Laboratory Animal Care International (File 000401), registered by the United States Department of Agriculture (51-F-0016) and maintains an assurance with the Public Health Service (A4149-01).

### Flow cytometry

Single-cell suspensions were prepared from thymus and spleens as per standard protocols. Hepatic lymphocytes were isolated from livers that were homogenized, filtered through nylon mesh and washed in PBS with 1 % FBS. Cells were then resuspended in 44 % Percoll (GE Healthcare Bio-Sciences AB, Uppsala, Sweden), underlaid with 66 % Percoll, and centrifuged for 20 min at 2000 rpm. Cells at the interface were collected and washed. Cells were stained, acquired on a FACSCantoII (Becton Dickinson) and analyzed with FlowJo (Treestar). Dead cells were excluded using the Fixable Viability Dye eFluor®506 (eBioscience). The following antibodies and their isotype controls conjugated to FITC, PE, PerCP-Cy5.5, PE-Cy7, APC, APC-Cy7 or Pacific Blue (from BD Biosciences, eBioscience or BioLengend) were used for staining: anti-CD4 (GK1.5), anti-CD8α (53–6.7), anti-TCRβ (H57-597), anti-CD1d (1B1), anti-Siglec-F (E50-2440), anti-Ly6G (1A8), anti-CD11c (N418), anti-CD11b (M1/70), anti-CD19 (6D5), anti-IFN-γ (XMG1.2), anti-IL-4 (11B11), anti-IL-13 (eBio13A) and anti-IL-17A (TC11-18H10.1). Anti-IL-17RB-APC (752101) and its isotype control were purchased from R&D Systems. PE- or APC- conjugated mouse CD1d tetramers loaded with glycolipid PBS-57 (CD1d-tet) were obtained from the tetramer facility of the US National Institutes of Health. In brief, cells were incubated with FC block and stained with antibodies, and then fixed with 2 % paraformaldehyde. For IFN-γ, IL-4, IL-13 and IL-17A intracellular staining, cells were permeabilized, stained and fixed using the BD Cytofix/Cytoperm kit (BD Biosciences). For PLZF and T-bet intracellular staining, cells were permeabilized and stained with anti-PLZF (D-9) (Santa Cruz Biotechnology, Inc.) plus FITC anti-mouse (BD Biosciences) and APC-conjugated anti-T-bet (eBio4B10) purchased from eBioscience, using the Foxp3 Staining Buffer kit (eBioscience).

### Bone marrow chimeras

For the BM chimera experiments, each recipient mouse received 2 × 10^6^ whole bone marrow cells from a single donor in 400 μl of PBS through i.v. injection. For the BM mixed chimera experiments, BM cells from two different types of donor mice were mixed at 1:1 ratio. Each recipient mouse received 9 × 10^6^ cells in 250 μl of PBS through i.v. injection. In all experiments, CD45 congenic markers were used to distinguish cells derived from the different sources. All BM chimeras were reconstituted for at least 7 weeks before analysis.

### In vitro PMA- and ionomycin-induced activation assay

For in vitro stimulation, thymocytes were cultured in T cell medium (RPMI 1640 with 10 % FBS, HEPES, penicillin and streptomycin, L-glutamine and 2-mer captoethanol) and stimulated for 5 h with phorbol 12-myristate 13-acetate (PMA) (50 ng/ml) and ionomycin (1 μM). For intracellular cytokine staining, Brefeldin A was added for the final 3.5 h.

### In vivo α-Galactosylceramide stimulation

Three micrograms of α-Galactosylceramide in 200 μl PBS were intraperitoneally injected into mice. Mice were sacrificed to obtain spleen 3 h after injection and splenocytes were stained for CD1d-tet, TCRβ, CD4, CD8α, IL-17RB and IL-4.

### AHR induction

The OVA/IL-25-induced AHR model was performed as previously described [29] with modification. Mice were immunized with 50 μg/2 mg i.p. OVA/alum twice (days 0 and 7), then treated with PBS or 2 μg IL-25 via i.p. at day 14 and finally challenged twice with 50 μg intranasal OVA (days 16 and 17). At day 18, analysis was performed. The mouse trachea was cannulated and lungs were lavaged with 1.5 ml cold PBS to obtain the BAL fluid from which, after centrifugation, cells were stained for flow cytometry with anti-TCRβ, anti-CD19, anti-Ly6G, anti-CD11b, anti-CD11c and anti-Siglec-F. The left lung lobe was inflated with 1 % low-melt agarose and fixed in formalin until embedded in paraffin to do sections that were stained with Hematoxylin and Eosin and Periodic acid Schiff. Photographs were taken with a Zeiss Axiophot microscope (lens: 10x Zeiss Plan Apo N.A. 0.32; lens: 25x oil Zeiss Plan Apo N.A. .80) with a Prog Res C14 digital camera attached to the scope. Camera software was Adobe Photoshop 6.0. Lymphocytes from the other lung lobes were isolated by digestion with RPMI medium containing collagenase II and DNase I followed by a lympholyte separation prior to staining for flow cytometry analysis.

### Statistics

Statistical significance was determined by the Student’s *t*-test.

## Results

### β-CAT-Tg mice show an exacerbated response to IL-25-dependent AHR induction

**Fig. 1:**
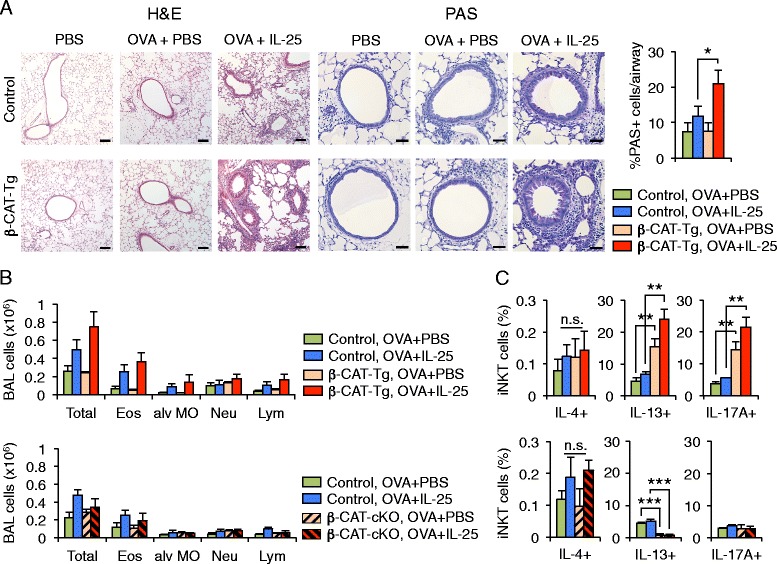
β-CAT-Tg mice show an exacerbated response to IL-25-dependent AHR induction. β-CAT-Tg, β-CAT-cKO and control mice were sensitized with OVA, treated with IL-25 or PBS and finally challenged with OVA to induce airway hyperreactivity (AHR). **a** Histological analysis of lung tissues with hematoxylin and eosin (H&E) and periodic acid Schiff (PAS) staining. Representative control and β-CAT-Tg sections are shown. H&E bars, 100 μm; PAS bars, 100 μm. Graph shows percentage of PAS-positive cells per total number of cells in the epithelium of the airway, counting 5–7 airways per lung section (mean ± sem). **b** Total cellularity (Total) and cell numbers of eosinophils (Eos), alveolar macrophages (alv MO), neutrophils (Neu) and lypmhocytes (Lym) present in bronchoalveolar lavage (BAL) fluid (*n* = 5, mean ± sem) from control and β-CAT-Tg *(top*) and control and β-CAT-cKO *(bottom).* **c** IL-4^+^, IL-13^+^ and IL-17A^+^ cell percentages of lung iNKT cells after IL-25-dependent AHR induction from control and β-CAT-Tg *(top)* and control and β-CAT-cKO (*bottom*) (*n* = 5, mean ± sem). AHR experiment was performed twice, with 3–5 mice per group in each independent experiment. * *P* < .05; ** *P* < .01

iNKT cells that migrate to the lung tissue are known to cause IL-25-dependent airway hyperresponsiveness (AHR). The receptor for IL-25 is IL-17RB, which is present in certain iNKT cell subsets [29]. IL-25 is both necessary and sufficient for Th2 cytokine production, eosinophilic airway inflammation and AHR [30–32]. To determine if enforced expression of β-catenin affects iNKT cell function we analyzed transgenic mice that express a proximal *Lck*-driven stabilized mutant form of β-catenin in thymocytes and T cells (β-CAT-Tg). We sensitized mice twice with OVA, along with treatment with IL-25 and finally challenged the mice intranasally with OVA. At the end of the protocol, we evaluated several hallmark characteristics of allergic lung inflammation. Histological analysis of lung sections from sensitized and challenged control and β-CAT-Tg mice showed typical features of allergic lung inflammation, which were particularly exacerbated in the β-CAT-Tg animals that were treated with IL-25 (Fig. 1a, left). Hematoxylin and eosin and Periodic acid Schiff (PAS) staining of the lung tissue revealed greater infiltration of immune cells and a significant increase in the number of mucus-producing cells in the lungs of IL-25 treated β-CAT-Tg mice compared to control mice (Fig. 1a, right). We quantified the immune cells infiltrating the airways by counting cells in the bronchoalveolar lavage fluid (BALF). We noticed an increase in all of these infiltrating cell types in the BALF from the challenged β-CAT-Tg mice (Fig. 1b, top). We wished to analyze the effect of the loss-of-function of β-catenin in AHR. To this end, we generated β-CAT^flox/flox^ CD4-Cre^+^ mice (referred to as β-catenin conditional knock-out, β-CAT-cKO). When the same experiment was performed in β-CAT-cKO mice, we did not see an increase in infiltration of cells compared to treated controls (Fig. 1b, bottom). These data suggest that β-CAT-Tg mice experience greater inflammation when challenged with OVA and IL-25 compared to control.

We further analyzed lung cell suspensions to detect the cytokine production by iNKT cells in the tissue. IFN-γ and IL-4 production was comparable in control, β-CAT-Tg and β-CAT-cKO mice challenged with OVA and IL-25 (Fig. 1c and data not shown). Strikingly, IL-13-expressing iNKT cells that have been specially implicated in IL-25-dependent AHR induction [29, 33, 34] were significantly enhanced in iNKT cells from lung cell suspensions from β-CAT-Tg mice (Fig. 1c, top). By contrast IL-13-producing iNKT cells in β-CAT-cKO mice were fewer and unresponsive (Fig. 1c, bottom). Finally, IL-17A was also largely produced by β-CAT-Tg iNKT cells (Fig. 1c). Thus, β-CAT-Tg IL-17RB^+^ iNKT cells contribute to a more robust allergic inflammatory response in the lungs of allergen-challenged β-CAT-Tg mice compared to control mice. The allergic response was particularly aggravated in CAT-Tg mice in the presence of IL-25 (Fig. 1a and c). These data demonstrate that in mice with enforced expression of β-catenin functional iNKT cells lead to a more robust response to IL-25-dependent AHR challenge, with all measured parameters of allergic inflammation enhanced, including higher iNKT production of Th2 and Th17 cytokines.

### β-Catenin expression regulates the differentiation of effector iNKT2 and iNKT17 cells

**Fig. 2:**
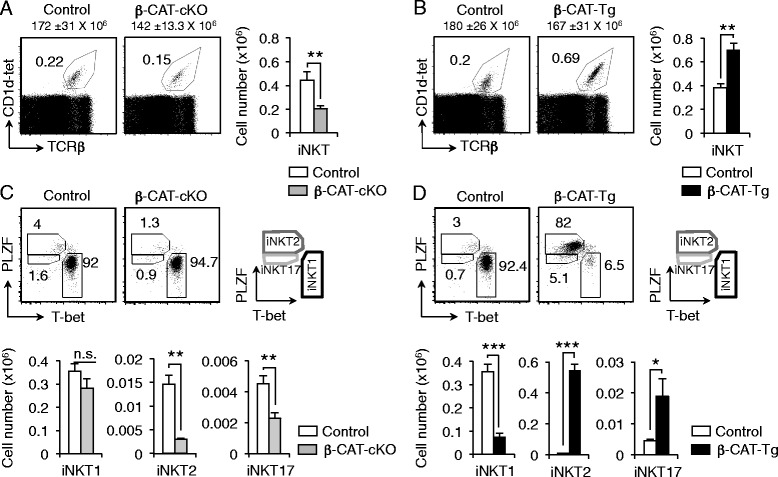
β-Catenin expression regulates the differentiation of effector iNKT2 and iNKT17 cells. **a**-**b** Frequency and total iNKT cell numbers from control and β-CAT-cKO mice (**a**) and from control and β-CAT-Tg mice (**b**) (n ≥ 4, mean ± sem). Numbers over dot plots refer to total thymocyte cell numbers. **c**-**d** Representative dot plots showing iNKT1, iNKT2 and iNKT17 populations among iNKT cells (*top*) and total numbers (*bottom*) from control and β-CAT-cKO mice (**c**) and from control and β-CAT-Tg mice (**d**). Gating strategies are shown in *right* (n ≥ 3, mean ± sem). * *P* < .05; ** *P* < .01; *** *P* < .001

To determine the specific role of β-catenin in iNKT cell development, we studied thymic iNKT cells in mice with T-cell conditional deletion or enforced expression of β-catenin. β-CAT-cKO mice showed similar proportions and total cell numbers of thymocytes (Fig. 2a, numbers on top of dot plots) and mature T cells compared to control mice (data not shown). However, β-CAT-cKO mice had a significant 50 % reduction in the number of iNKT cells (Fig. 2a). β-CAT-Tg mice, on the other hand, presented a remarkable increase in the number of thymic iNKT cells compared to control mice (Fig. 2b). In light of these data, we conclude that β-catenin expression is limiting for the generation of thymic iNKT cells.

Analysis of expression of transcription factors PLZF and T-bet in iNKT cell populations from control, β-CAT-cKO and β-CAT-Tg mice showed that the frequency and number of iNKT2 and iNKT17 cells was significantly affected by β-catenin expression. Specifically iNKT2 and iNKT17 cells, but not iNKT1 cells, accounted for the major decrease in β-CAT-cKO and the greatest increase in β-CAT-Tg mice (Fig. 2c-d). We conclude that β-catenin expression is required for the differentiation and expansion of iNKT2 and iNKT17 cells.

### β-Catenin regulates cytokine production by iNKT cells

**Fig. 3:**
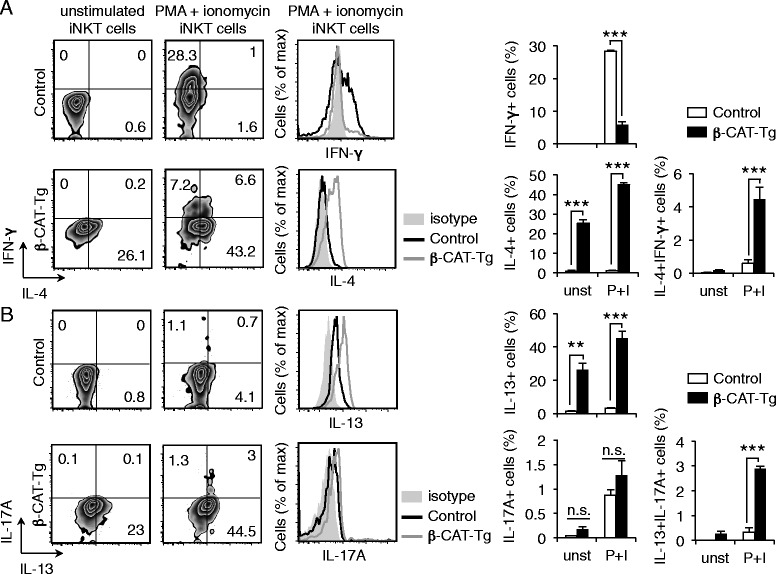
β-Catenin regulates cytokine production by iNKT cells. Frequency of IFN-γ- and IL-4-producing (**a**) and IL-13- and IL-17A-producing (**b**) iNKT cells from control and β-CAT-Tg mice in in vitro unstimulated or PMA + ionomycin stimulated iNKT cells. *Left*, representative experiment and *right,* graph showing mean ± sem, *n* = 3. * *P* < .05; *** *P* < .001

iNKT cells acquire functional capabilities in the thymus as part of the developmental program. To determine if β-CAT-Tg iNKT cells were functionally skewed towards a Th2/Th17 phenotype, we cultured β-CAT-Tg thymocytes in vitro with PMA (50 ng/ml) and ionomycin (1 μM) for 5 h and then assayed IFN-γ, IL-4, IL-13 and IL-17 production by thymic iNKT cells. The results showed that the frequency of IFN-γ-producing iNKT cells from β-CAT-Tg mice was significantly reduced compared to iNKT cells from control mice (Fig. 3a). Strikingly, a much higher percentage of iNKT cells from β-CAT-Tg mice expressed IL-4, compared to control iNKT cells, even in unstimulated conditions (Fig. 3a). Likewise, we found enhanced production of IL-13 and also IL-13 + IL-17A+ double-producers by β-CAT-Tg iNKT cells compared to control iNKT cells (Fig. 3b). We conclude that enforced expression of β-catenin promotes a higher frequency of Th2- and Th17-like iNKT cells. Importantly, we note that in the absence of stimulation approximately 26 % β-CAT-Tg thymic iNKT cells produced IL-4 compared to 0.6 % control thymic iNKT cells (Fig. 3a). These data demonstrate that β-catenin promotes the acquisition of effector functionality in developing iNKT cells in the thymus.

### β-Catenin regulation of iNKT2 and iNKT17 cells is maintained in the periphery

**Fig. 4:**
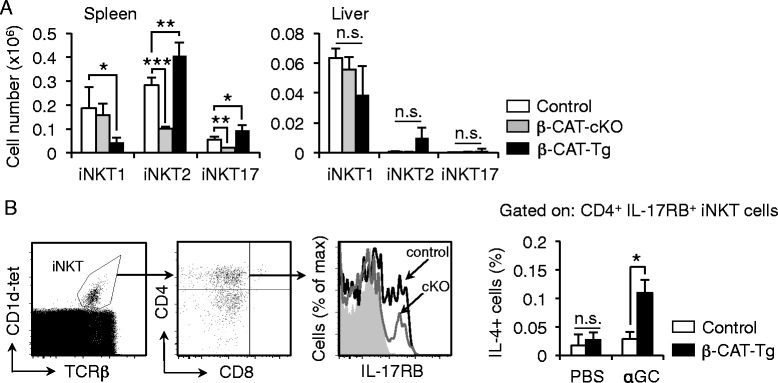
β-Catenin regulation of iNKT2 and iNKT17 cells is maintained in the periphery. **a** Total cell numbers of iNKT1, iNKT2 and iNKT17 cells from control, β-CAT-cKO and β-CAT-Tg mice in spleen and liver (*n* = 3, mean ± sem). **b** Gating strategy for CD4^+^ IL-17RB^+^ iNKT cells is shown in *left. Right*, frequency of IL-4-expressing CD4^+^ IL-17RB^+^ iNKT cells from spleens of control and β-CAT-Tg mice 3 h after injection with PBS or 3 μg α-Galactosylceramide (αGC). * *P* < .05; ** *P* < .01; *** *P* < .001

We then examined the distribution of iNKT cells in the periphery in the β-catenin mutant mice. We observed that spleen and liver from β-CAT-cKO mice showed a specific reduction of the iNKT2 and iNKT17 cells in the spleen, and β-CAT-Tg spleens had reduced iNKT1 and increased iNKT2 and iNKT17 cells (Fig. 4a). Analysis of liver from control, β-CAT-cKO and β-CAT-Tg mice showed comparable numbers of iNKT1 cells (Fig. 4a). By contrast, the frequency of cytokine-producing cells was significantly diminished in β-CAT-cKO mice (Fig. 4a). We conclude that altered iNKT cell development in β-CAT-cKO and β-CAT-Tg mice is reflected in the β-catenin-dependent skewing towards iNKT2 and iNKT17 cells in the distribution of these cells in the periphery.

To further test the efficacy of induced response of Th2-like iNKT2 cells expanded in β-CAT-Tg mice we tested cytokine production in vivo. To address this we administered 3 μg α-galactosylceramide (αGC) intraperitoneally to β-CAT-Tg and control mice and 3 h post-injection analyzed cytokine production by splenic iNKT cells. αGC is known to activate iNKT cells in vivo [1, 11, 35]. Importantly, we found that αGC-stimulated CD4+ IL-17RB+ iNKT cells (gated as in Fig. 4b, left, and mainly iNKT2 cells [29]) in β-CAT-Tg mice produced high levels of IL-4 (some IL-4+ IFNγ + cells were noted but are not included in this analysis) (Fig. 4b, right). Therefore, we conclude that enforced expression of β-catenin promotes Th2-biased cytokine production by iNKT2 cells in response to stimulation in vivo.

### Enhanced generation of β-catenin-dependent iNKT2 and iNKT17 cells is cell autonomous

**Fig. 5:**
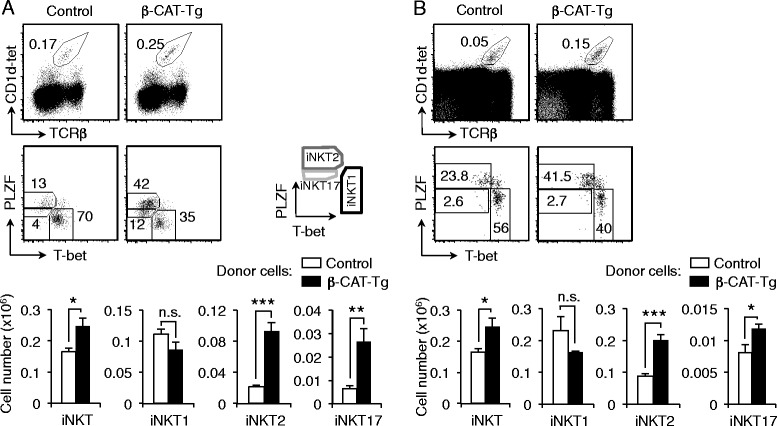
Enhanced generation of β-catenin-dependent iNKT2 and iNKT17 cells is cell autonomous. **a** Flow cytometry of a bone-marrow chimera experiment analyzed after 7–8 weeks, in which CD45.1+ recipient C57BL/6.SJL mice were irradiated and 2 million cells isolated from bone marrow were i.v. injected from CD45.2+ donors, either control or β-CAT-Tg mice. *Top,* total iNKT cells gated on CD45.2+ cells. *Middle*, iNKT1, iNKT2 and iNKT17 cells within the iNKT populations. *Bottom*, number of total iNKT, iNKT1, iNKT2 and iNKT17 cells from donor control and donor β-CAT-Tg cells (n ≥ 8, mean ± sem). **b** Flow cytometry of CD45.1 and CD45.2 on thymic cells of mixed bone-marrow chimeras containing CD45.1+ recipient C57BL/6.SJL cells, CD45.1 + CD45.2+ donor control cells and CD45.2+ donor β-CAT-Tg cells. iNKT cells from both donor cells (*top*) and iNKT1, iNKT2 and iNKT17 cells within the iNKT populations *(Middle*). Representative data out of three mice analyzed. *Bottom*, number of total iNKT, iNKT1, iNKT2 and iNKT17 cells from donor control and donor β-CAT-Tg cells (*n* = 3, mean ± sem). Mice were analyzed 7 weeks after mixed bone-marrow chimeras were performed. * *P* < .05; ** *P* < .01; *** *P* < .001

To determine if the effect of enforced β-catenin expression on iNKT cell generation was cell intrinsic, we generated chimeric mice using either control or β-CAT-Tg cells as bone marrow donor cells. Analysis of chimeric mice showed that the frequency and numbers of iNKT2 and iNKT17 cells was significantly greater when the donor bone marrow was derived from β-CAT-Tg mice compared to control mice (Fig. 5a). Furthermore, mixed chimeric mice generated with bone marrow cells derived from wild-type control (CD45.1 + CD45.2+) and from β-CAT-Tg (CD45.2+) transferred into irradiated wild-type mouse acting as recipient (C57BL/6.SJL, CD45.1+) also showed significantly increased generation of iNKT2 and iNKT17 cells (Fig. 5b). Thus, this data demonstrates that β-catenin controls differentiation of effector iNKT2 and iNKT17 cells in a cell-intrinsic manner.

## Discussion

In this study we demonstrate that β-catenin regulates the differentiation of iNKT cells to iNKT2 and iNKT17, but not iNKT1, effector subsets. Conditional deletion of β-catenin in iNKT cell-precursors impaired differentiation of iNKT2 and iNKT17, but not iNKT1 cells. By contrast, enforced expression of β-catenin promoted the generation of iNKT2 cells and iNKT17 cells without affecting generation of iNKT1 cells. The effect of enhanced β-catenin expression in iNKT cell precursors to promote development of iNKT2 and iNKT17 cells was cell-autonomous. Importantly, we show that β-CAT-Tg iNKT cells acquired the ability to produce enhanced levels of IL-4 and IL-13 during development that augmented lung inflammation in a mouse model for asthma.

Cytokine production by iNKT1, iNKT2 and iNKT17 effector subsets has been shown to be controlled by transcription factors T-bet, GATA3 and RORγt that also regulate IFN-γ, IL-4 and IL-17 production by T helper cells [8–10]. However, transcriptional control of differentiation of iNKT0 cells to iNKT1, iNKT2 and iNKT17 subsets has remained to be defined. This report shows that β-catenin deficiency resulted in a profound decrease in iNKT2 and iNKT17 subsets of iNKT cells whereas iNKT1 cells developed normally. By contrast, enforced expression of β-catenin promoted the development of iNKT2 and iNKT17 cells. It was important to note that the majority of iNKT cells in the thymus of C57BL/6 mice were iNKT1 cells and the observation that enforced expression of β-catenin altered the pattern to iNKT2 and iNKT17 cells suggests that β-catenin may be a major factor in the distinct pathways that critically direct differentiation of iNKT effector subsets. These observations are congruent with the observation that a cohort of genes (including IL-7R, IL-17RB, Cxcr3, Cxcr6, Ccr4, c-Myc, c-Myb, Bcl11b, Zbtb16, Egr2, Id3, Slamf1 and Slamf6) that have been previously implicated in iNKT cell development were up-regulated in mice with enforced expression of β-catenin and diminished in expression in cells with deletion of β-catenin. Unlike effector CD4 T cells, iNKT cells acquire functional capabilities during the differentiation and maturation process. It was interesting to note that β-CAT-Tg iNKT cells developed the capacity to produce enhanced levels of IL-4, which was reminiscent of the report that TCF1 and β-catenin promote expression of GATA3 and IL-4 in T helper 2 cells [36]. Thus, we posit that β-catenin-dependent gene expression regulates the differentiation and functional maturation of iNKT2 and iNKT17 cells.

Finally, our results obtained from the allergic lung inflammation mouse model may be of some clinical importance. Although the precise role for the different human iNKT subsets in the lung and peripheral blood has yet to be clearly defined, correlative studies suggest that iNKT cells are involved in exacerbations of asthma [37, 38]. Furthermore, IL-25 has been reported to be increased in asthmatic humans [39]. Patients with high IL-25- and IL-17RB-containing airway epithelial cells present a more prominent Th2 reaction and respond better to inhaled corticosteroids treatment [40]. We hypothesize that iNKT2 cells may also be increased in IL-25-high asthmatic patients facilitating the generation of “Th2-high” asthma, as opposed to a severe, neutrophil-mediated, steroid-insensitive disease. Modula ting the numbers of iNKT cells may be beneficial in reducing asthma symptoms and exacerbations. Targeting novel pathways or molecules involved in iNKT differentiation, such as β-catenin as we have described here, could be a new approach. In conclusion, this study shows that β-catenin expression regulates the differentiation of iNKT cells that augment lung inflammation associated with asthma and provides insights that may be of use in designing regimens to limit at least one type of lung inflammation.

## Conclusions

In conclusion data presented in this paper indicate that β-catenin regulates the differentiation of iNKT cells to iNKT2 and iNKT17, but not iNKT1, effector subsets. We propose that iNKT2 and iNKT17 cells promote asthma and posit that modulating the development and function of these cells provides novel means to control of lung inflammation.

## Abbreviations

iNKT: Invariant Natural Killer T
DP: double positive thymocytes
TCR: T-cell receptor
iCD8: innate-like CD8
IL: Interleukin
AHR: Airway hyperresponsiveness
IFN-γ: Interferon gamma
αGC: α-galactosylceramide
